# Supplementary material for: Status exploration and analysis of regional hospice and palliative care networks in Germany: A protocol for a mixed-methods study
Source: PLoS One. 2023 Jun 2;18(6):e0286583. doi: 10.1371/journal.pone.0286583 (PMC10237382; doi:10.1371/journal.pone.0286583)
Supplement: Attachment — Submitted filename: 2023_04_11 Response to Reviewers_HOPAN.docx [file pone.0286583.s003.docx]

MHH – Allgemeinmedizin – OE 5440 – 30623 Hannover

**Institut für Allgemeinmedizin und Palliativmedizin**

**Direktor: Prof. Dr. med. Nils Schneider**

Sekretariat, OE 5440

Frau Gomm

Telefon:	0511 532-2744

Fax:	0511 532-162744

allgemeinmedizin@mh-hannover.de

Carl-Neuberg-Straße 1

30625 Hannover

www.mh-hannover.de/allgemeinmedizin.html

11. April 2023

Dear editors,

dear reviewers,

thank you for giving us the opportunity to submit a revision of our manuscript “Status exploration and analysis of regional hospice and palliative care networks in Germany: A protocol for a mixed-methods study” (PONE-D-22-32383).

We would like to thank the reviewers for their affirmative comments and valuable suggestions. We have considered them carefully in the revision of the manuscript.

| The authors write about "palliative care", also in the title. I believe it should be made clear, that this is rather "specialist palliative care" or "joint general and specialist palliative care". Please consider rewording after discussion in your author group. | In the introduction, it was added that the networks include all providers of general and specialised palliative care in the outpatient and inpatient setting. |
| --- | --- |
| The definition of "hospice" is differing between different international health care settings. You may consider clarifying this not also in the introduction, but even though this is iterative, in the discussion. Please feel free to ignore this suggestion, if your author group (at least the key- authors) agrees to do so after thourough discussion. | In the introduction, the term "hospice" in Germany was explained and the hospice care structures were described, referring to the S3- Leitlinie “Palliativmedizin für Patienten mit einer nicht-heilbaren Krebserkrankung”. |

We graciously thank you for your time in evaluating the revised version of our manuscript.

Yours sincerely

Dr. phil. Sven Schwabe
